# An Unusual Presentation of Pectoralis Major Pyomyositis Presenting as Septic Arthritis of the Shoulder: A Case Report and Review of the Literature

**DOI:** 10.3390/diseases6040100

**Published:** 2018-11-10

**Authors:** Ahmed Saad, Shafiq Shahban, Tarek Elgamal

**Affiliations:** Heartlands Hospital, Birmingham B9 5SS, UK; shafiqshahban@hotmail.com (S.S.); tarek.elgamal@heartofengland.nhs.uk (T.E.)

**Keywords:** pyomyositis, septic arthritis, shoulder, pectoralis muscle

## Abstract

Pyomyositis is a relatively rare condition and often requires a low index of suspicion. We present a case of an otherwise fit and well woman who had pyomyositis of the pectoralis major muscle and presented as an acute septic arthritis of the shoulder. We present the conundrums that arose in arriving at this diagnosis, and how we successfully managed this condition through our multidisciplinary approach. We urge all clinicians to bear in mind this potential diagnosis, even in those patients not deemed to be immunocompromised.

## 1. Introduction

Pyomyositis is a rare condition and is often regarded as a disease which, being tropical in nature, is thus more common in parts of Africa and the South Pacific. Despite this, its incidence in the Western World has increased gradually in recent years [1].

Pyomyositis describes as a subacute deep bacterial infection involving skeletal muscle, most frequently affecting the musculature of the lower limbs (quadriceps, hamstrings, gluteal muscles and iliopsoas). In most cases, the aetiology of pyomyositis is unclear; however, risk factors thought to be associated with this condition include immunosuppression, strenuous exercise and direct muscle trauma [2]. Pyomyositis, as in this case, can often catch the clinician by surprise, because it may not be an obvious differential diagnosis, and/or patients can present with a variety of non-specific symptoms, including those which may make one consider septic arthritis [3].

We report a rare case of spontaneous extensive pectoralis major pyomyositis, presenting as a septic arthritis of the shoulder, in a patient with no identifiable risk factors. We highlight the perplexing presentation of our patient and the appropriate investigations undertaken that led to the diagnosis, as well as the importance of early management and intervention.

## 2. Case Report

Mrs. ET, a 54-year-old woman, was admitted to our unit with a two-week progressive history of dull aching pain over her right shoulder. Her symptoms had progressively worsened over the few days prior to admission. The pain was associated with fever and rigours and was aggravated by minimal shoulder joint movement. She was not diabetic, and there was no history to suggest immunosuppression, with hypertension being the only past medical history of note. Additionally, there was no history of any trauma preceding her symptoms.

On examination, she had a core body temperature of 37.9° Celsius with otherwise normal physiological parameters (i.e., within normal range). There was no history of a recent illness, and on systematic review her only complaint was a painful right shoulder. On inspection of the right shoulder, there was no obvious erythema, with a small amount of swelling and localised temperature change over the right shoulder joint. Her shoulder tenderness was vague and spanned from her scapula through to her sternoclavicular joint. She had a reduced amount of shoulder movement (in every direction) and was specifically tender on arm adduction against resistance.

**Figure 1:**
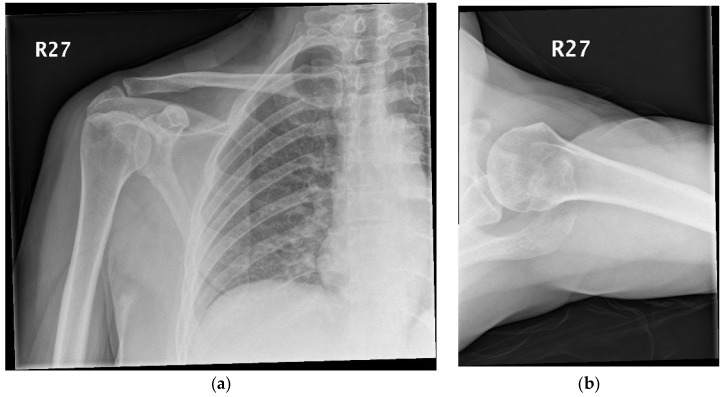
(**a**,**b**) Radiographs of the right shoulder.

Laboratory investigations revealed raised inflammatory markers, with a white cell count (WCC) of 9.62 × 10^9^/L, neutrophils of 8.73 × 10^9^/L, and a C-reactive protein (CRP) of 52 mg/L. Radiographs of the right shoulder were obtained (Figure 1a,b), and were unremarkable.

Mrs. ET had a recorded episode of pyrexia in the department, and at that point had additional blood tests taken for culture and sensitivity. Equally, at this point, septic arthritis of the shoulder could not be excluded and thus two attempts at shoulder aspiration were made (with an anterior and then a posterior approach), with no success.

Blood cultures from Mrs. ET had grown *Staphylococcus aureus* which was found to be sensitive to flucloxacillin, and so, after discussion with the infectious diseases (ID) team, the patient was commenced on intravenous antibiotics. An echocardiogram was also performed soon after and was found to show no evidence of an infective endocarditis.

The following day, Mrs. ET’s episodic spikes in body temperature had not settled and at that moment in time, despite a dry tap from the shoulder, the working diagnosis was a septic arthritis of the shoulder. Consequently, preparations were made for Mrs. ET to head to theatre for an arthroscopic (or potentially open) aspiration and washout of the shoulder joint.

**Figure 2:**
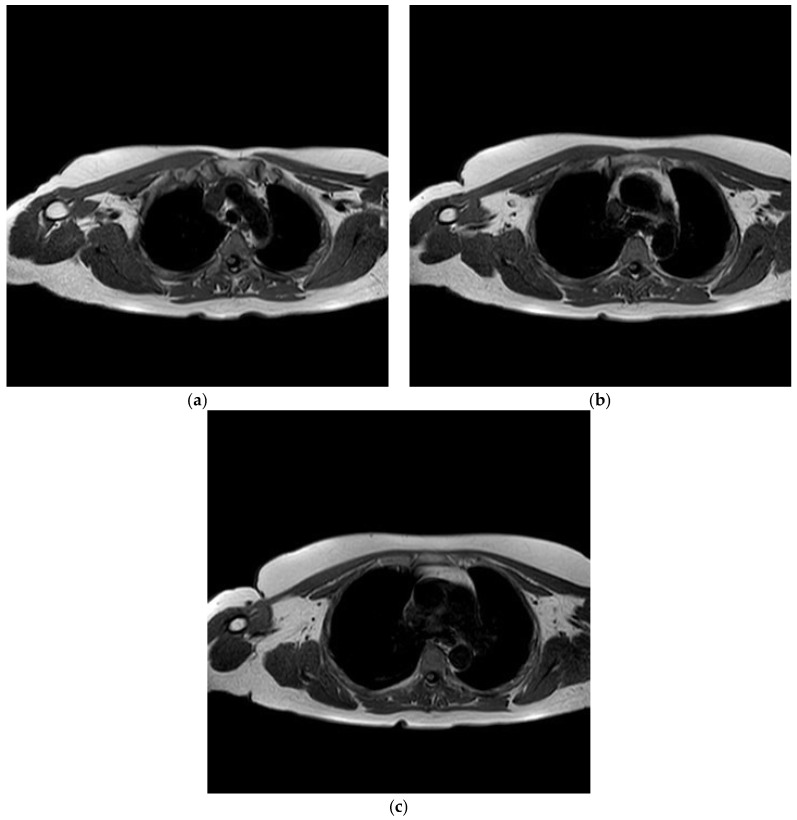
(**a**–**c**) Axial images demonstrating right pectoralis major pyomyositis.

Prior to the operation, an MRI (magnetic resonance imaging) scan was requested to assess/confirm the presence of a collection within the joint. The MRI scan went on to demonstrate no collection within the shoulder joint; however, inflammation involving the right pectoral muscle approximately 8.8 cm in circumference was observed, suggesting pectoralis major pyomyositis (Figure 2a–c).

Mrs. ET did not proceed to surgical intervention and, after further discussions with the ID team, was given a two-week course of intravenous antibiotics (Flucloxacillin 2 g, four times a day). Over the course of her hospital stay, the CRP fell to 19 mg/L and the patient’s episodes of pyrexia settled completely. After this two-week course, Mrs. ET was prescribed a four-week course of oral Clindamycin (300 mg, four times a day).

At six-week follow up, the patient’s symptoms had almost fully resolved, with near full range of movement in the shoulder joint and a further drop in her CRP to 5 mg/L. A repeat MRI scan of the shoulder confirmed complete resolution of the pyomyositis. After a course of shoulder physiotherapy, Mrs. ET did not require any further antibiotics and was discharged from follow up.

## 3. Discussion

Pyomyositis is a disease that is predominantly seen in tropical countries, characterised by suppuration of bacteria within skeletal muscles. In recent years, there has been a gradual increase in the number of cases in non-tropical areas, especially the United Kingdom, with four reported cases in the literature, manifesting as single or multiple abscesses [4,5,6,7].

Pyomyositis is commonly seen in immunocompromised patients and conditions associated with it include HIV, diabetes, leukaemia and chronic renal failure. In certain cases, pyomyositis has been seen to be linked to trauma and/or vigorous exercise [2,8]. As in this case, *Staphylococcus aureus* is the organism most commonly responsible for causing this condition, cultured in up to 90% of cases in tropical areas and 75% in non-tropical countries [1].

Early diagnosis of this condition is critical for saving the muscle tissue and also the life of the patient, and so having a low index of suspicion for this is key. Patients with this condition, certainly when muscle close to a joint is involved, can present and mimic a septic arthritis, which can make diagnosing this condition difficult. What made things more difficult in this patient’s case was that she was not immunosuppressed and had no history of immunosuppression.

Diagnosis of pyomyositis involves having a low index of suspicion and working through a series of investigations. There are no set criteria; together, clinical judgement and physiological parameters as well as biochemical investigations can help to rule in (or out) such a diagnosis [9,10]. Mazur et al. found MRI to be 97% sensitive for diagnosing acute musculoskeletal infections [11]. Through this case we demonstrated how the MRI scan was vital to help to rule out a collection within the shoulder joint and to prevent unnecessary surgery. Equally, the MRI aided us in making our diagnosis and helped us to monitor the regression in the size of the collection.

Pyomyositis presents as a subacute condition and has different stages in its progression [12]. Early diagnosis is important to not only initiate treatment, but also to investigate for complications of septicaemia, namely bacterial endocarditis. A multidisciplinary approach is integral to the success of managing such patients. Given how well Mrs. ET responded to intravenous antibiotics, surgery, in the form of abscess drainage and/or debridement of necrotic tissues, was not required [13].

## 4. Conclusions

Pyomyositis is a rare disease; however, it should be included in the differential diagnoses when patients present with vague symptoms of discomfort around a joint with signs of sepsis. Despite this condition being more prevalent amongst immunosuppressed patients, it is important to consider this when dealing with patients who may not be immunocompromised, as early diagnosis will lead to a more favourable outcome.
